# Real-world cost-effectiveness of cetuximab in the third-line treatment of metastatic colorectal cancer based on patient chart review in the Netherlands

**DOI:** 10.1186/s13561-018-0197-3

**Published:** 2018-07-17

**Authors:** Carin A. Uyl-de Groot, Elisabeth M. van Rooijen, Cornelis J. A. Punt, Chris P. Pescott

**Affiliations:** 1 Erasmus School of Health Policy & Management/institute for Medical Technology Assessment, Erasmus University Rotterdam, P.O. Box 1738, 3000 DR Rotterdam, the Netherlands; 2 Erasmus School of Health Policy & Management, Erasmus University Rotterdam, Rotterdam, the Netherlands; 3 Department of Medical Oncology, Academic Medical Center, University of Amsterdam, Amsterdam, the Netherlands; 4 Global Evidence & Value Development, Merck KGaA, Darmstadt, Germany

**Keywords:** Cost effectiveness, Metastatic colorectal cancer, Cetuximab, *KRAS*, Third-line treatment

## Abstract

**Objective:**

To assess the cost effectiveness of cetuximab in third-line treatment of patients with *KRAS* wild-type (wt) metastatic colorectal cancer (mCRC) in routine clinical practice compared with best supportive care (BSC).

**Methods:**

Patients (*n* = 287) with *KRAS* wt mCRC treated with cetuximab or BSC in eight hospitals in the Netherlands between 2009 and 2012 were included in our real-world study. Outcome measures were costs per life-year (LY) and costs per quality-adjusted LY (QALY) gained. A Markov model was developed, and a time horizon of four years was applied. Outcomes were calculated from Kaplan-Meier survival curves from patient-level data and literature. Direct medical costs were estimated in all centers (2013 values), and incremental cost-effectiveness ratios (ICERs) were calculated. Results were discounted, and a probabilistic sensitivity analysis was performed.

**Results:**

Administration of cetuximab in third-line treatment of mCRC resulted in a gain of 0.29 LYs and 0.25 QALYs compared with BSC. In the four-year study period, average discounted healthcare costs were €36,637 in the cetuximab group vs. €3648 in the BSC group. The discounted ICERs of cetuximab vs. BSC in the real-world setting were €114,907and €133,527 per LY and QALY gained, respectively.

**Conclusions:**

Results of this cost-effectiveness analysis showed that third-line treatment with cetuximab for patients with *KRAS* (exon 2) wt mCRC offered clinical benefits at additional cost. The real-world ICERs were in line with those of previously published cetuximab and panitumumab cost-utility models.

## Background

Cancer is the most frequent cause of mortality worldwide, with an estimated 8.2 million cancer-related deaths in 2012. Colorectal cancer (CRC) is one of the most prevalent cancers, the third most commonly diagnosed cancer in men and the second in women, with 746,000 and 614,000 new cases annually, respectively. Additionally, it is the third-leading cause of cancer-related mortality worldwide, with 373,500 deaths annually [1].

The incidence of CRC is expected to increase in the coming decades. Therapies containing oxaliplatin, irinotecan, and biologics have improved overall survival (OS). In the mid-1970s, 5-year OS rates were 50%, and this rate has since increased to 66% [2]. Biologic agents targeting vascular endothelial growth factor, its receptor, or epidermal growth factor receptor (EGFR) are the mainstay of biologic treatment options.

Two drugs targeting EGFR have been licensed, cetuximab and panitumumab [3, 4]. For these drugs, reimbursement within the Dutch healthcare system was regulated under an “expensive drugs policy.” This policy allowed for conditional reimbursement, contingent on delivering evidence of real-world drug utilization and demonstrating associated cost-effectiveness in Dutch daily practice after 4 yrs. In this paper we present the results of a real-world study concerning cost-effectiveness of cetuximab in third-line treatment of patients with *KRAS* (exon 2) wild-type (wt) metastatic CRC (mCRC) compared with best supportive care (BSC).

## Methods

### Design

We performed a retrospective observational study of patients with *KRAS* (exon 2) wt mCRC who received cetuximab or BSC between 2009 and 2012 in eight hospitals in the Netherlands. The participating medical centers were academic and general hospitals located in the central and western parts of the Netherlands. Approval for the study was obtained from medical ethics committees and the board of directors where medical ethical review was not required.

Patients were eligible for this study if they had been diagnosed with mCRC, experienced treatment failure with two prior lines of anticancer drug therapy, had been tested for *KRAS* (exon 2) mutation, and had received cetuximab monotherapy or BSC. Information on all patients who were tested for *KRAS* status was obtained from each hospital, irrespective of what treatment they had received. All patients diagnosed with mCRC were included in the study. Patients diagnosed with *KRAS* wt mCRC and treated with cetuximab were identified through the hospital pharmacy. Their patient data were grouped into either a treated group, which included all patients treated with cetuximab, or a control group consisting of patients tested for a *KRAS* (exon 2) status but who had not subsequently received cetuximab. The inclusion date for this study was the date that *KRAS* (exon 2) status was confirmed. Clinical data were collected from the date on which the *KRAS* (exon 2) status test had been ordered. Patients were followed up until either death or the end of data collection, in June 2013.

No direct comparison could be made between patient groups receiving either cetuximab or BSC, due to differences in baseline characteristics, including *KRAS* (exon 2) status. This was considered in a matching protocol. Two sub-analyses were performed to correct for possible differences. Firstly, only patients who were treated in the third line were compared. Secondly, pairs were created matched on nine variables: gender, age, presence and severity of comorbidities, location of primary tumor, number of organs with metastasis, *KRAS* (exon 2) mutation status, and number and length of prior treatments. Age was matched using a difference calculation. If the difference between the age of the cetuximab-treated patient and the BSC-treated patient was 5 yrs or less, this was considered a match. A similar strategy was used for length of treatment; a difference in treatment duration of 30 days or less was considered a match.

Performance status and laboratory test results were not included in the matching technique due to the large amount of missing information. However, the presence of comorbidity, the Charlson Comorbidity Index, and the number of organs with metastases were considered an adequate proxy for these variables. A patient who was treated with cetuximab had to match a patient treated with BSC on these three criteria as well as four of the remaining six criteria to be considered a match. No matching control could be found for three cetuximab-treated patients, and they were thus excluded from the analysis. When multiple matching cases could be identified, the first matching (previously unmatched) BSC-treated patient available was chosen as a control.

Data on disease progression could not be obtained from hospital records for patients receiving BSC as these data are not routinely collected when no anticancer therapy is administered. Outcomes from Karapetis et al. [5] were used to estimate effectiveness of BSC in daily practice because this study compared third-line cetuximab plus BSC vs. BSC alone. By combining the extrapolated hazard ratio from the real-world cetuximab group with the hazard ratio from Karapetis et al., we calculated the hazard ratio of the real-world BSC group. This provided an effect estimate for the time from treatment start to disease progression. Although clinical trial patients may have more favorable baseline characteristics than a real-world population, we assumed that the effect ratio would be similar. Overall survival (OS) curves were drawn using the Kaplan-Meier method. Statistical significance was assumed if the *p* value was below 0.05. Outcome measures were costs per life-year (LY) gained and costs per quality-adjusted LY (QALY) gained.

### Data collection

Data were collected from case report forms for 287 patients. Patient characteristics included age, gender, Eastern Cooperative Oncology Group performance status, laboratory values (e.g., hemoglobin, liver and kidney panels, and carcinoembryonic antigen), *KRAS* (exon 2) status, and the test used to determine mutation status. Information on OS was available for all patients, while progression-free survival (PFS) data were available only for those patients treated with cetuximab. If a patient had died, the date of death was recorded; if disease had progressed, the date of the diagnostic test (e.g., blood work and radiological test) was recorded. Data on drug utilization were collected, including details regarding cetuximab administration, such as planned dose and actual dose administered. For reported adverse events, grade, duration, and drug management measures were recorded. Furthermore, for cost analysis, resource use data, such as hospital days, outpatient visits, laboratory tests, medical procedures, and other drugs, were collected for both patient groups.

Unit costs for the various types of resources used were expressed in 2013 euros and were taken from a previous cost-effectiveness study in mCRC and inflated to reflect 2013 costs [6]. When unit costs were not available, they were derived from the Dutch Manual for Costing, the Dutch Healthcare Authority, or the literature (Table 1) [7, 8].

**Table 1:** Main unit costs

Unit description	Cost, 2013 €
Oncology inpatient day	518
Intensive care day	2201
Oncology daycare visit	177
Oncology outpatient visit	104
Consultation by phone	15
Laboratory service by day	45
Emergency room visit	184
X-ray	52
Computed tomography scan	223
Magnetic resonance imaging	258
Radionuclear scan	193
Positron emission tomography scan	1485
Ultrasound	79
Colonoscopy	438
Port-A-Cath insertion	399
Radiotherapy (fraction)	97

### Model structure

**Fig. 1:**
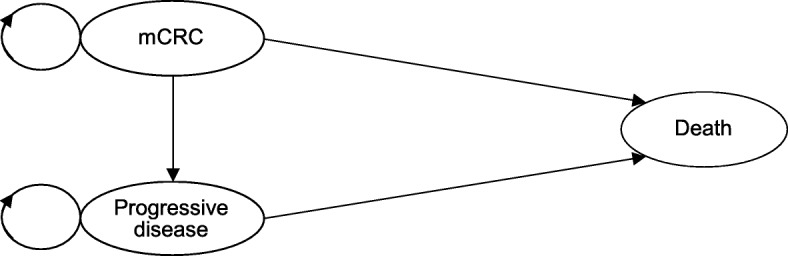
Model structure. mCRC, metastatic colorectal cancer

A Markov model was developed to simulate patient transition through three distinct health states: mCRC before disease progression, progressive disease, and death (Fig. 1). All patients started in the progression-free health state (“mCRC before disease progression”) and could transition to either “progressive disease” or “death.” From “progressive disease,” patients could transition only to “death.” Cycle length was defined as 2 weeks because cetuximab is given weekly [6, 9, 10].

Costs and effects were discounted at 4% and 1.5%, respectively, in accordance with Dutch guidelines [11]. The time horizon for the base-case analysis was set as 4 yrs to allow all effects and costs to accumulate. This was considered adequate to represent a lifetime horizon because median OS times of 8.1 and 9.5 months have been reported with cetuximab in the third-line treatment of *KRAS* (exon 2) wt mCRC [5].

### Sensitivity analysis

To reflect the uncertainty regarding the parameters and observed variables used in the model, a probabilistic sensitivity analysis (PSA) was performed. A PSA quantifies model outcomes assuming parameter values are point estimates from a probability distribution. In a PSA, for each variable, a point estimate is drawn from its respective distribution, providing insight into the uncertainty surrounding parameter values (Table 2).

**Table 2:** Distributions used per parameter in the probabilistic sensitivity analysis

Parameter	Type of distribution	Source
Transition probabilities	Weibull distribution	Real-world data and RCT
Costs	γ distribution	Real-world data
Utilities	β distribution	RCT

*RCT* randomized controlled trial

## Results

### Comparison of baseline characteristics

Baseline characteristics for age, performance status, and duration of treatment show statistically significant differences between groups (Table 3). Patients receiving cetuximab had a better performance status than patients receiving BSC. The total duration of treatment was significantly longer for cetuximab patients. The duration of active treatment prior to start of cetuximab or BSC did not differ between groups. The differences between the two groups, especially regarding performance status, suggest that any comparison between the two groups must be interpreted with caution.

**Table 3:** Baseline characteristics of patients in outcomes study

Patients previously treated with two treatment lines		Cetuximab group (*n* = 23)	BSC group (*n* = 35)	*P* values
Male		60%	65%	NS (*p* = 0.16)
Age, median (years)		64	60	*p* < 0.05
PS	0	26%	6%	*p* < 0.05
	1	17%	11%	
	2	9%	3%	
	3	–	–	
	4	4%	–	
	Unknown	43%	80%	
Location	Colon	48%	54%	NS (*p* = 0.9)
	Rectum	35%	31%	
	Rectosigmoid	13%	9%	
	Unknown	4%	6%	
Charlson Index^a^	6	35%	63%	NS (*p* = 0.1)
	7	35%	17%	
	8	17%	11%	
	9	–	6%	
	10	9%	–	
	Unknown	4%	3%	
Mean total treatment duration (days)		398	264	*p* < 0.05
Mean treatment duration prior to BSC or cetuximab (days)		268	264	NS (*p* = 0.9)

*BSC* best supportive care, *NS* not significant, *PS* performance status

^a^Severity as scored with the Charlson Index; the presence of mCRC leads to an automatic score of 6 as baseline

### Base-case analysis

**Fig. 2:**
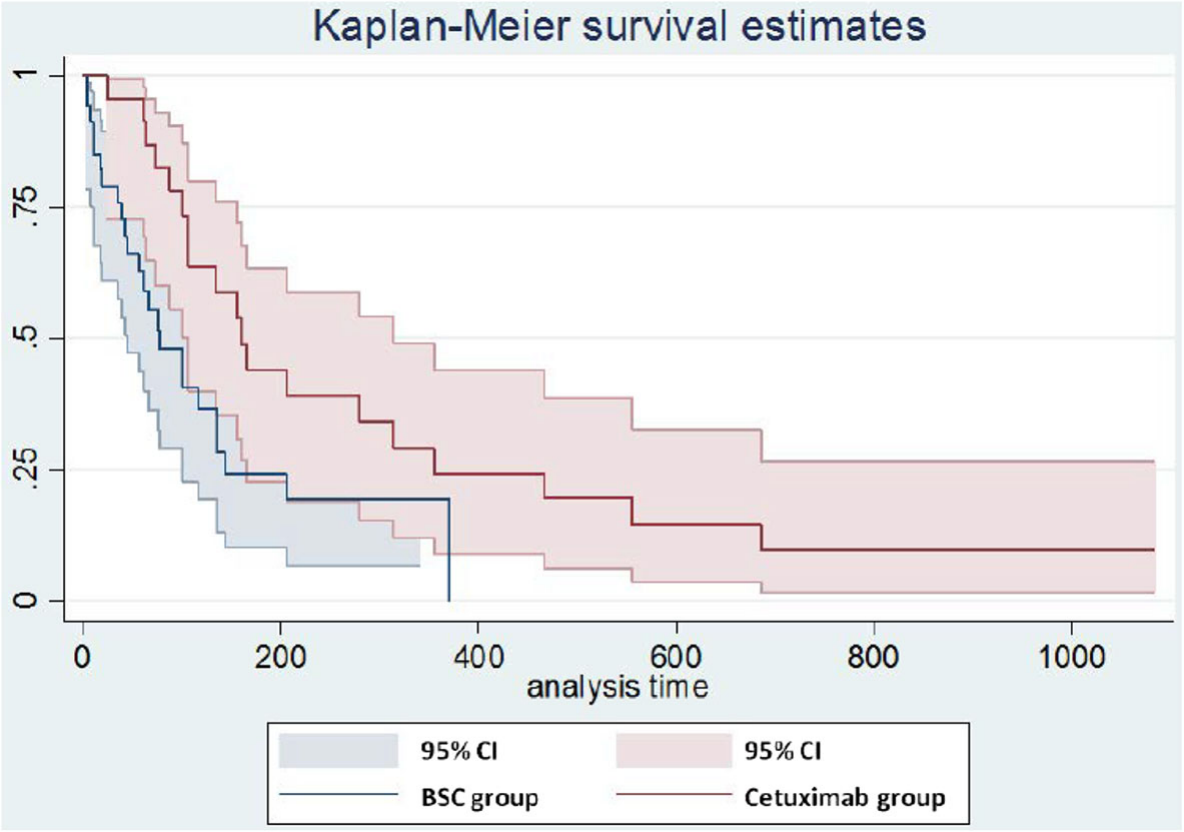
Kaplan-Meier estimates of survival for patients treated in the third line with cetuximab or BSC. BSC, best supportive care

Based on Kaplan-Meier estimates for patients in the third line of treatment, the median OS was 5.2 months and 2.5 months in the cetuximab and BSC groups, respectively (*p* < 0.05) (Fig. 2). The results of the base case are shown in Table 4. The mean survival of patients treated with cetuximab was 0.61 LYs (7.32 months) compared with 0.32 LYs (3.84 months) in the BSC group, resulting in survival gains favoring the cetuximab group of 0.29 LYs (approximately 3 months). The quality-adjusted result for the cetuximab group was 0.48 QALYs. The result for the BSC group was 0.24 QALYs, a gain of 0.25 QALYs (after rounding). The discounted effects were nearly identical.

**Table 4:** Results of model analysis (deterministic and probabilistic sensitivity analysis)

	LY (95% CI)	QALY (95% CI)	Cost, € (95% CI)
Deterministic analysis (undiscounted)			
Cetuximab + BSC	0.61	0.48	37,146
BSC	0.32	0.24	3678
Incremental	0.29	0.25	33,468
ICER	115,690	134,495	
Deterministic analysis (discounted)			
Cetuximab + BSC	0.61	0.48	36,637
BSC	0.32	0.23	3648
Incremental	0.29	0.25	32,989
ICER	114,907	133,527	
PSA (undiscounted)			
Cetuximab + BSC	0.61 (0.57–0.64)	0.48 (0.45–0.51)	36,915 (26,773–48,716)
BSC	0.32 (0.30–0.34)	0.24 (0.22–0.25)	3639 (2520–4982)
Incremental	0.29 (0.25–0.33)	0.25 (0.21–0.28)	33,276 (22,720–45,027)
ICER	116,030 (80,417–158,009)	134,777 (92,521–184,072)	
PSA (discounted)			
Cetuximab + BSC	0.61 (057–0.64)	0.48 (0.45–0.51)	36,410 (26,407–48,053)
BSC	0.32 (0.30–0.34)	0.24 (0.22–0.25)	3609 (2498–4942)
Incremental	0.29 (0.24–0.33)	0.25 (0.21–0.28)	32,801 (22,394–44,397)
ICER	115,248 (79,861–156,946)	133,812 (92,521–184,072)	

*BSC* best supportive care, *ICER* incremental cost-effectiveness ratio, *LY* life-year, *PSA* probabilistic sensitivity analysis, *QALY* quality-adjusted life-year

For patients treated with cetuximab, the average total costs were €37,146 per patient; for patients in the BSC group, these were €3678 before discounting. The main cost drivers were drug costs, costs of hospitalizations, and additional chemotherapy.

The resulting discounted incremental cost-effectiveness ratios (ICERs) amounted to €114,907 per LY gained and €133,527 per QALY. The undiscounted results are presented in Table 4.

### Probabilistic sensitivity analysis

**Fig. 3:**
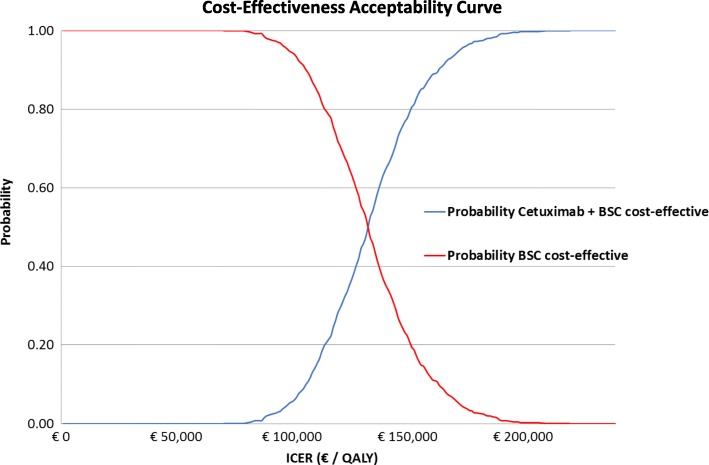
Cost-effectiveness acceptability curve of the discounted model analysis. BSC, best supportive care; ICER, incremental cost-effectiveness ratio; QALY, quality-adjusted life-year

**Fig. 4:**
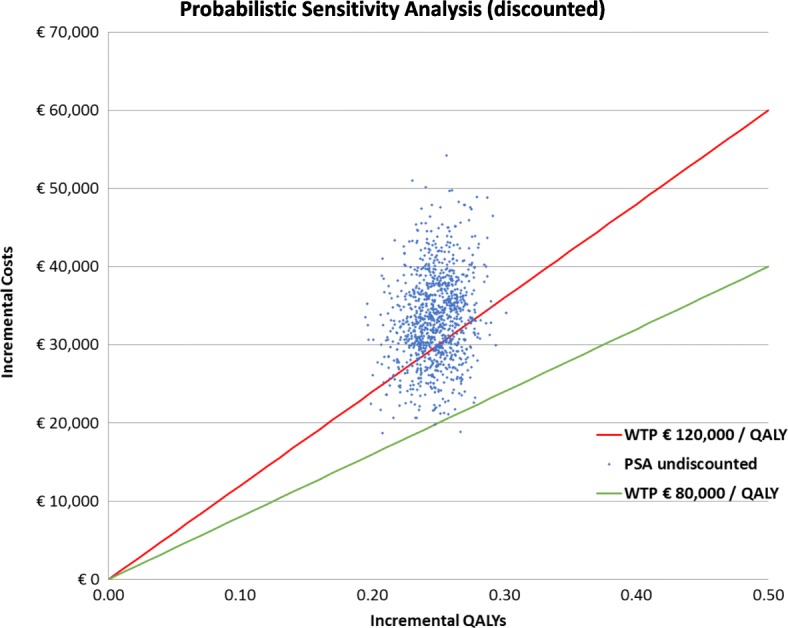
Scatter plot of ICERs from the undiscounted probabilistic sensitivity analysis. PSA,  probabilistic sensitivity analysis; QALY, quality-adjusted life-year; WTP, willingness to pay

When uncertainty was addressed in the PSA, the mean discounted ICER was €133,812/QALY (95% CI, €92,521-€184,072; Table 3). The associated cost-effectiveness acceptability curve for the discounted cost per QALY showed that at a value of €100,000/QALY, the probability of cetuximab being a cost-effective strategy was approximately 10%; at a threshold value of €150,000/QALY, the probability was approximately 80% (Fig. 3). The scatter plot of ICERs from the PSA is shown in Fig. 4. This figure shows that in all PSA runs, cetuximab resulted in QALY gains, however, at the same time, also in considerable additional costs. The mean undiscounted ICER of the PSA was €138,296 and yielded a range for the ICER of €92,521 to €184,072.

## Discussion

To our knowledge, this study is the first of its kind to calculate a real-world ICER for cetuximab in this setting. The results led to an ICER of €133,527/QALY (95% CI, €92,521-€184,072/QALY).

Our study has some limitations. First, clinical records did not contain sufficient information to determine PFS in the BSC group and, furthermore, no quality-of-life data were available in the medical records. To overcome this shortcoming, data from randomized controlled trials (RCTs) were used to populate the model in cases in which real-world data were lacking. RCT data were extrapolated and combined with real-world data to populate the BSC arm of the model, and we also applied quality-of-life data derived from the same RCT [12]. In the RCTs and in real-world data, treatment with cetuximab compared with BSC was associated with almost a doubling of the median OS and PFS among patients with *KRAS* wt tumors.

In an RCT, patients are treated according to a predefined protocol and have more active follow-up than patients treated under real-world conditions. Additionally, RCTs often apply stricter inclusion and exclusion criteria (e.g., patients with good performance status and few comorbidities), whereas in the real world, older patients with a multitude of comorbidities and poorer condition are also eligible [13]. In the RCTs, more patients had colon and/or rectum cancer. In the real-world data, there were relatively more rectosigmoid cancers. Concerning the performance status, the availability of recorded data was too limited to draw a conclusion about differences between the states of patients. Further, the RCT patients in general received more adjuvant treatments than the real-world patients did. As a result, treatments given may have been less intensive and the outcome of these treatments may have been worse than those seen in RCTs [13].

Our study design did not allow for randomization of patients. However, by matching patients from the control group to patients in the cetuximab group, we corrected for differences between the two groups at baseline. This way, known possible confounders were corrected for. However, whether eligible patients may receive cetuximab in daily practice depends on physician and patient choice; the rationale is not always provided in the medical record. As a result, we could not directly correct for these possible confounders.

Further, we assumed similarity of the effect ratio between the clinical trial and routine daily practice. The literature has shown that when patient characteristics were similar between RCT data and real-world data, assuming similarity in effect size yielded a valid approximation of relative effectiveness outcomes [14]. In our study, these differences, were marginal and tested in the PSA.

Cost data collection was conducted from a hospital perspective rather than a societal perspective [11]. A hospital perspective was chosen for this study because a societal perspective was not feasible. Societal costs were therefore not collected; however, due to the patients’ extended survival, the main sources of nonmedical costs in the Netherlands are often productivity loss or additional medical expenses unrelated to the condition under study. The population in this study had a mean age of 63 years, while the mean age of retirement in the Netherlands in 2009 was < 63 years [15]. Productivity losses, or potential gains, will therefore be minimal in this population. Additionally, because the survival of patients with mCRC often does not extend beyond 2 years, even with optimal treatment, it is unlikely that medical expenses unrelated to the primary condition would yield a large difference between the treated and BSC groups in this study. However, travel costs are a type of cost that may differ between these two groups as patients treated with cetuximab require weekly hospital visits to receive treatment, whereas patients receiving only BSC will visit less regularly. The differences in costs may be limited because cetuximab is a treatment that can be given at nearly any hospital, and the average distance to a hospital in the Netherlands is only 7 km [16].

Additionally, home care provided to a patient by a spouse, family member, or friend may lead to loss of productivity for the caregiver, and both longer survival and side effects in patients receiving cetuximab may result in more loss of productivity for their caregivers than would occur for patients receiving BSC only. We did not estimate the impact of these costs on the ICER.

For the cost analysis, different sources were used. Some limitations may be introduced by drawing data from different sources; however, the rationale for using different sources is inherent to the study design. Some data could not be obtained from hospitals, such as the resource use of BSC. However, Dutch guidelines provide a valid and reliable framework for such resource use and associated costs. Furthermore, the impact of the different costs has been studied by conducting a PSA.

Scientific articles on the cost utility of cetuximab monotherapy are scarce [17]. Hoyle et al. [18] reported an ICER of €127,300/QALY for cetuximab compared with BSC based on an indirect treatment comparison. In that study, utility values were adapted based on general population values, and resource use was partially based on patients treated for breast cancer.

Tappenden et al. [19] performed a threshold analysis from a UK National Health Service perspective. The conclusion of this analysis was that it was unlikely that an ICER of < €40,200/QALY could be achieved with cetuximab, even when a rule was applied that would mandate treatment to be stopped if no effect was observable after 6 weeks. Mittmann et al. [20] calculated the cost effectiveness of cetuximab treatment in Canada and reported an ICER for cetuximab of €209,729/QALY compared with BSC, substantially higher than the result of €134,777/QALY found in this study. This difference may be due to resource use in the analysis by Mittmann et al. being determined through trial data rather than daily practice. Additionally, the Mittmann publication references a higher per-mg cost of cetuximab than the drug cost used in our calculations.

Publications on the cost-effectiveness of panitumumab, a biologic with a similar marketing license, are scarce as well [17]. Hoyle et al. [18] reported an ICER for panitumumab in the United Kingdom of €250,580/QALY compared with BSC. The only other report on panitumumab cost-effectiveness was published only as an abstract by Graham et al. [21]. The ICERs reported were for the comparison of panitumumab plus BSC vs. BSC alone as third-line treatment of patients with *KRAS* (exon 2) wt mCRC. The ICERs were €51,314/LY gained and €59,440/QALY gained. These estimates were based on RCT data only, and the analyses were performed in a Dutch setting. Because the report is in abstract form only, the information presented was limited [17].

An upper bound of €80,000/QALY has been suggested for the Netherlands for the most severe illnesses [22]. Recently, however, there has been discussion on whether drugs used during the last stages of illness (end-of-life products) should be held to a different standard, as is the case in the United Kingdom [23, 24]. If so, a higher threshold might be more appropriate for such drugs. Additionally, the panitumumab indication was changed based on an analysis published by Douillard et al. [25], whereas the cetuximab indication was changed based on the re-analysis of the OPUS and CRYSTAL studies with regard to *NRAS* and *KRAS* exons 3 and 4 mutations [26, 27].

## Conclusions

Our research showed an incremental effectiveness for use of cetuximab in third-line treatment of *KRAS* (exon 2) wt mCRC of 0.25 QALYs over BSC in Dutch daily practice. The resulting discounted, deterministic ICERs were €114,907/LY and €133,527/QALY. Drug costs were identified as the main cost driver.

## Abbreviations

BSC: Best supportive care
CRC: Colorectal cancer
EGFR: Epidermal growth factor receptor
ICER: Incremental cost-effectiveness ratio
LY: Life-year
mCRC: Metastatic colorectal cancer
OS: Overall survival
PFS: Progression-free survival
PSA: Probabilistic sensitivity analysis
QALY: Quality-adjusted life-year
RCT: Randomized controlled trial
wt: Wild type
